# Elements of Surgical Diagnosis

**Published:** 1885-03

**Authors:** 


*Elements of Surgical Diagnosis.* By A. PEARCE GOULD, F.R.C.S. Pp. 562. London: CASSELL & CO. 1884.

It may be prejudice, but we confess to a want of sympathy with a surgeon who describes himself in the title-page of his work as "Surgeon to the London Temperance Hospital." If such a surgeon acts up to the spirit of his propaganda, then we believe that his patients suffer, and that he is not to be trusted as an enlightened teacher of the practice of surgery. His temperance tenets, however, may not interfere with his capacities for diagnosis.

There is undoubtedly room for an English work on Surgical Diagnosis; and to this work, as one of the excellent series of manuals issued by Cassell and Co., we were prepared to give the same hearty welcome that we have given to its fellows. We are sorry that we cannot unreservedly do so. In many respects the book is worthy of commendation. It is full; it is clearly and tersely written, and it shows evidence of much reading—we had almost said of too much reading. At all events, there is evidence of a want of clinical insight and practical knowledge, which occasionally results in a curious want of the sense of proportion in estimating the values not only of the symptoms, but also of the facts on which they are founded. We could fill several pages of extracts to illustrate our criticism; but one or two sentences must suffice. Speaking of the organisation of blood-clot, he says (p. 47): "If a wound be wholly or in part filled up with a blood-clot, and this clot do not soften and flow away, but remains firm and fixed; and if after several days a thin dry layer of it separate and show a soft delicate cicatrix below, it is called *organisation of, or more correctly in, a blood-clot*. This is the process which has long been familiar in small wounds as *healing under a scab*." We would not dare to express an opinion, derived as it would be merely from his written language, that Mr. Gould has never seen an organising blood-clot; but we are almost certain that he has seen enough to prove that clot organisation is not "*the* process" of "healing under a scab." If he has not, we must recommend a visit to a vaccination station, or a place of entertainment frequented by Irishmen, where he will see other processes of healing under a scab.

On page 129 we meet this statement: "The presence of local emphysema or of a pneumatocele proves a *fracture of ribs*." Imagine the consternation of a senescent College of Surgeons examiner when confronted by such a remark from the lips of a young student! The glib facility with which the unpractised student will jumble up the most ordinary and unimportant with the most rare and serious symptoms is sometimes startling to the experienced man: he would find plenty of sentences of this sort in the book before us.

We doubt if this work will be much patronised by students. In the main it is above them. The balance of symptomatology is not evenly maintained. The arrangement of facts is neither easy nor natural; and there is too much overlapping of statements. Still, we believe that Mr. Gould has here collated the materials of a very useful work. With a free use of the pruning-knife, a more exact appreciation of admitted facts, and a rearrangement of its contents, we believe that the book might have a future before it.

